# Programmed cell death-1, PD-1, is dysregulated in T cells from children with new onset type 1 diabetes

**DOI:** 10.1371/journal.pone.0183887

**Published:** 2017-09-06

**Authors:** Hector M. Granados, Andrew Draghi, Naomi Tsurutani, Kyle Wright, Marina L. Fernandez, Francisco A. Sylvester, Anthony T. Vella

**Affiliations:** 1 Department of Pediatrics, Texas Tech Health Science Center, El Paso, Texas, United States of America; 2 Department of Immunology, University of Connecticut Health Center, Farmington, Connecticut, United States of America; 3 Department of Pediatrics, Connecticut Children’s Medical Center, Hartford, Connecticut, United States of America; 4 Department of Pediatrics, University of North Carolina at Chapel Hill, Chapel Hill, North Carolina, United States of America; Children's Hospital Boston, UNITED STATES

## Abstract

**Background:**

Programmed death cell 1 (PD-1) is an inhibitor of T cell activation and is also functionally linked to glycolysis. We hypothesized that PD-1 expression is defective in activated T cells from children with type 1 diabetes (T1D), resulting in abnormal T cell glucose metabolism.

**Methods:**

In this pilot study, we enrolled children with new onset T1D within 2 weeks of diagnosis (T1D), unaffected siblings of T1D (SIBS), unaffected, unrelated children (CTRL), children with new onset, and untreated Crohn disease (CD). We repeated the assays 4–6 months post-diagnosis in T1D (T1D follow up). We analyzed anti-CD3/-CD28-stimulated peripheral blood mononuclear cells (PBMC) subsets for PD-1 expression by flow cytometry at baseline and after 24 h in culture. We measured cytokines in the culture medium by multiplex ELISA and glycolytic capacity with a flux analyzer.

**Results:**

We enrolled 37 children. T cells derived from subjects with T1D had decreased PD-1 expression compared to the other study groups. However, in T1D follow-up T cells expressed PD-1 similarly to controls, but had no differences in PBMC cytokine production. Nonetheless, T1D follow up PBMCs had enhanced glycolytic capacity compared to T1D.

**Conclusions:**

Activated T cells from T1D fail to upregulate PD-1 upon T-cell receptor stimulation, which may contribute to the pathogenesis of T1D. T1D follow up PBMC expression of PD-1 normalizes, together with a significant increase in glycolysis compared to T1D. Thus, insulin therapy in T1D children is associated with normal PD1 expression and heightened glycolytic capacity in PBMC.

## Introduction

Type 1 Diabetes (T1D) is an autoimmune disease caused by autoreactive CD4 and CD8 T cells that destroy insulin-producing β-cells in the pancreas, resulting in hyperglycemia and its complications [1]. Our understanding of the mechanisms that underlie T cell dysregulation in humans with T1D is limited. T cell responses are controlled by the balance of activating and restraining regulatory pathways. Co-stimulatory and check point inhibitory molecules play important roles in self-tolerance. Of these, the CD80/CD86/CD28 B7 co-stimulatory pathway is one of the best understood [2]. CD80 and CD86 can bind to either an activation (CD28) or inhibitory (CTLA-4) receptor on T cells, determining its functional phenotype. Programmed cell death-1 (PD-1) and its ligand PD-L1 are also part of the B7 family [3]. PD-1 is expressed on activated T cells and inhibits T cell activation after binding to PD-L1[4]. The level of PD-1 expression and the extent of engagement of PD-1 by its ligands regulate the threshold for T cell activation and amount of cytokines produced[5]. These functions of PD-1: PD-L1 in immune cell activation are only beginning to be understood in T1D[6]. Guleria et. al. reported that PDL1 blockade accelerated diabetes onset in the NOD mice. Their study suggests that PDL1 may prevent autoimmune diabetes by limiting the expansion of CD4^+^ and CD8^+^ autoreactive T cells [7]. In the non-obese diabetic (NOD) mouse for example, PD-1 suppresses infiltration of autoreactive T cells in the pancreas, suggesting a critical protective role for PD-1 in T1D in mice [8].

In adults with long standing T1D, Tsutsumi et. al., reported that PD-1 gene expression in peripheral CD4^+^ T cells from was significantly lower than in healthy control subjects[9]. We therefore hypothesized that PBMCs of children with *newly diagnosed* T1D fail to upregulate PD-1 upon stimulation and that decreased PD-1 expression is associated with over-expression of pro-inflammatory cytokines by PBMCs. We aimed to analyze the expression of PD-1 in resting and stimulated PBMCs in 5 study groups: children with new onset T1D (T1D), their unaffected siblings (SIBS), unaffected, unrelated controls (CTRL) and children with chronic inflammation without autoimmunity (newly diagnosed, untreated Crohn disease—CD), and the same T1D 4–6 months post diagnosis (T1D follow up). Given the recently reported relationship between glycolytic capacity, cytokine production and PD-1 expression in murine T cells by Chang et al.[10], we examined indicators of glycolysis in PBMCs from the 5 study groups, and correlated them with PD-1 expression and cytokine potential plus skewing.

## Methods

### Study population

The Institutional Review Board (IRB) of Connecticut Children’s Medical Center and the IRB at the University of Connecticut Health Center approved all study procedures. Written informed consent was obtained from the next of kind, caretaker, or guardian on behalf of the minor/children, also assent of minor was obtained. Children with diabetic ketoacidosis or with known comorbidities (asthma requiring systemic steroids, celiac disease, Addison disease or autoimmune thyroid disease) were excluded, as well as children taking oral corticosteroids in the past 3 months.

### Subject enrollment

A study coordinator (MLF) offered the study to children with T1D during the first 2 weeks following the diagnosis, while children evaluated for CD were offered the study prior to diagnostic endoscopy (when blood samples can be obtained from an IV placed for sedation). Unaffected siblings of children with T1D were invited to participate during the same visit as their affected sibling. Unaffected, unrelated controls evaluated for abdominal pain were enrolled at the hospital’s pediatric gastroenterology practice. A nurse obtained a venous blood sample from each patient. Blood was collected in 4 tubes, 3 tubes (3 mL each) with buffered sodium citrate (BD, Franklin Lakes, NJ) for PBMCs isolation, and one tube (10 mL) with sodium heparin (BD, Franklin Lakes, NJ) to obtain plasma (20 mL total blood collection from each participant). After collection blood was double-bagged, put in a Styrofoam container with cool packs and transported to our laboratory at the University of Connecticut Health Center (typically a 15–20 min drive).

### Sample processing

For isolation of PBMCs each blood sample was diluted 1:1 (v/v) with phosphate-buffered saline (PBS, pH 7.4), layered over Lympholyte H (Cedarlane Laboratories, Ontario, Ca), and centrifuged at 800 X *g* for 20 min at 25°C. The buffy coat was aspirated, cells washed twice in RPMI and frozen at -86°C in 85% fetal bovine serum and 15% DMSO or used immediately for subsequent analyses (please see below).

### Culture conditions and T cell activation method

Culture medium consisted of RPMI-1640 supplemented with 10% fetal bovine serum and antibiotic/antimycotic at 1X (all from Gibco, Life Technologies, Carlsbad, CA). T cell activation was performed with a Human T-cell Activation/Expansion kit containing anti-CD3 and anti-CD28 antibodies (Miltenyi Biotec, Bergisch Gladbach, Germany), used according to the manufacturer’s protocol. Briefly, anti-biotin MACSiBead particles were resuspended thoroughly and 25 μL transferred onto a 1.8 mL Eppendorf tube. Fresh medium (200 μL) was added and then centrifuged at 300 X *g* for 5 min, the supernatant aspirated and the pellet resuspended in 100 μL fresh medium. Cells where suspended in 900 μL of medium, and added to the MACSiBead particles for a total volume of 1000 μL. The resulting mixture was distributed at a density of 250,000 cells/well in a 96-well plate (Greiner Bio-one, Monroe, NC) and incubated at 37°C for 24 h in a 5% CO_2_ atmosphere.

### Flow cytometry detection of PD-1 expression in PBMCs

**Fig 1:**
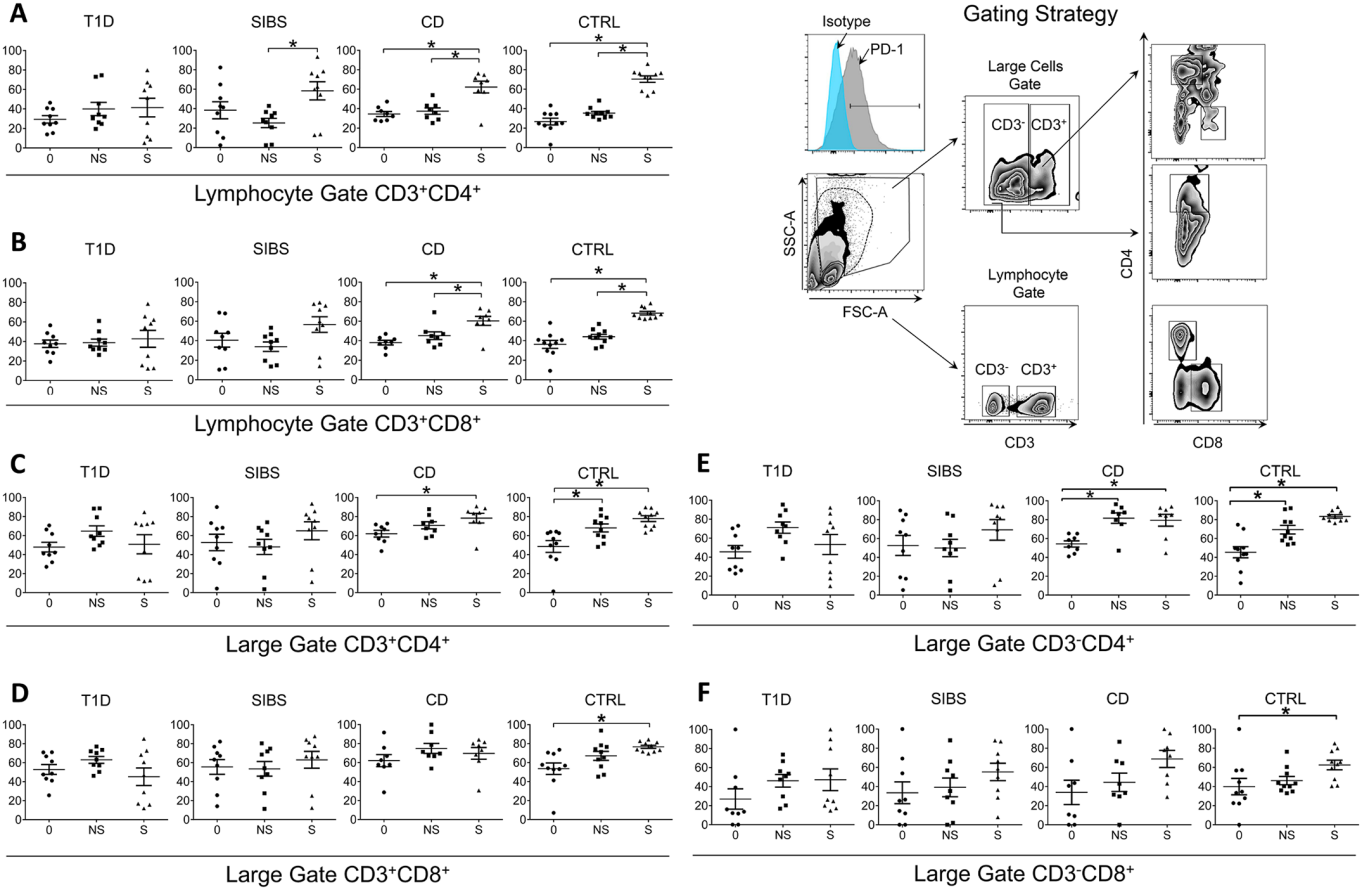
T Cells from T1D fail to upregulate PD-1 upon stimulation. Freshly isolated PBMCs were stained with antibodies against CD3, CD4, CD8, and PD-1 and analyzed by flow cytometry. Lymphocytes were identified by forward and side scatter and interrogated for CD4 and CD8 expression (bottom of right panel, lymphocyte gate). Another gate with higher side scatter identified larger cells that include monocytes and activated T cells (top of right panel, large gate). Mean percentage expression of PD-1 was compared by one-way ANOVA on each of the PBMC subpopulations (**A-F**). Percentage PD-1 surface expression in PBMC subsets is shown, at time zero (0) and after 24 h in culture with (S) and without (NS) stimulation (* indicate p < 0.05).

To assess the cell-surface expression of PD-1 on distinct PBMCs subpopulations by flow cytometry, PBMCs were counted and placed in 96-well plates and washed twice with 200 μL/well cell staining buffer (Hank’s buffered salt solution, 3% fetal bovine serum and 0.1% sodium azide, 200 μL/well). Fc receptors were blocked with FcR Block (Miltenyi Biotec GmbH) in 50 μL staining buffer, 3 min at 25°C. Cells were stained with antibodies to surface markers of interest (all with corresponding isotypes) in a total volume of 100 μL: CD3-VioBlue (Miltenyi Biotec GmbH), CD8-VioGreen (Miltenyi Biotec GmbH), CD4-PerCPCy5.5 (Peridinin chlorophyll protein complex -BioLegend, San Diego, CA), anti-PD-1-APC (eBioscience, San Diego, CA). After washing and resuspending, cell surface fluorescence was analyzed using a BD LSR II Flow Cytometer (BD Biosciences, San Diego, CA). Gates of interest were defined using Flow-Jo Software V.10 (Tree Star, Inc. Ashland, OR) and encompassed either lymphocytes or large cells (Fig 1). In each gate the number and percentage of cells that expressed surface PD-1 was examined at baseline and after 24 h in culture with and without stimulus. Mean percentage expression of PD-1 was compared by one-way ANOVA on each of the PBMCs subpopulations among the 4 study groups and T1D Follow up.

### Cytokine analysis by multiplex assay on supernatants obtained from PBMCs

The supernatants obtained after the culture of PBMCs with and without stimulus from subjects of the 4 study groups and T1D follow up were tested on a multiplex assay cytokine array in triplicate (Q-Plex Human Cytokine—Screen, Quansys Biosciences, Logan, UT). Cytokines assessed included IL-2, IL-4, IL-6, IL-12p70, IL-17, IL-23, IFN-γ and TNF-α, selected based on their importance on T1D pathogenesis, or importance on T cell activation[11].

### Extracellular flux analysis in PBMCs

The index of glycolytic function, approximated by extracellular acidification rate (ECAR), as measured in XF media (Glycolytic Stress Base Medium containing 2 mM L-Glutamine, Seahorse Bioscience, MA) under basal conditions and in response to glucose (10 mM). Cells were counted and distributed at a density of 300,000–400,000 cells/well, all in triplicate. Glycolytic capacity was examined after treatment with 2.5 μM oligomycin, an inhibitor of ATP synthase, and glycolytic reserve with 1 M, 2-Deoxy-D-Glucose [2-DG]. Measurements were performed on a XF-96 Extracellular Flux Analyzer (Seahorse Bioscience) [10].

### Statistics

All statistical analyses were performed using GraphPad Prism 6 statistical software (GraphPad Software, Inc. La Jolla, CA). Skewed data were first log10-tranformed to obtain normal distributions. We compared means among groups by one-way ANOVA.

## Results

### Demographic information

Baseline subject’s characteristics are depicted in Table 1. There was no significant difference in mean age among the study groups, but males predominated among children with T1D.

**Table 1:** Demographic information.

Characteristic	T1D	T1D Follow up	SIBS	CTRL	CD
**Number of subjects**	9	8	10	10	8
**Age (y, mean ± SD)**	11.6 ± 3	11.2 ± 2.9	10.6 ± 3.6	13.1 ± 2.8	12 ± 3.5
**Gender**	8 M, 1 F	7 M, 1 F	6 M, 4 F	5 M, 5 F	5 M, 3 F

### PD-1 surface expression is reduced in PBMCs from T1D, but recovers 4–6 months post-diagnosis

In the lymphocyte gate, the percent expression of PD-1was not significantly higher in stimulated (S) CD3^+^CD4^+^ T cells of T1D compared to the other 3 study groups, especially CD and CTRL (Fig 1 panel A, *see NS to S within each group*). A similar pattern was observed in CD3^+^CD8^+^ T cells (Fig 1 panel B). Thus, T1D CD3^+^ CD4^+^ or CD8^+^ lymphocytes failed to upregulate PD-1 to levels seen in CTRL or even compared to CD. In the higher side and forward scatter gate (“large gate”), activated T cells (both CD4^+^ and CD8^+^) from T1D did not increase PD-1 expression upon stimulation either (Fig 1 panel C or Fig 1 panel D). Regarding heterogeneous CD3^-^CD4^+^ cells from the “large gate” that expressed PD-1 (Fig 1 panel E), subjects with T1D did not upregulate PD-1 in culture, in contrast to CD and CTRL, which increased PD-1 expression despite the lack of T-cell receptor, suggesting a bystander effect of anti-CD3/anti-CD28 stimulation on these cells. CD3^-^CD8^+^ cells, however, did not upregulate PD-1 upon stimulation (Fig 1 panel F).

**Fig 2:**
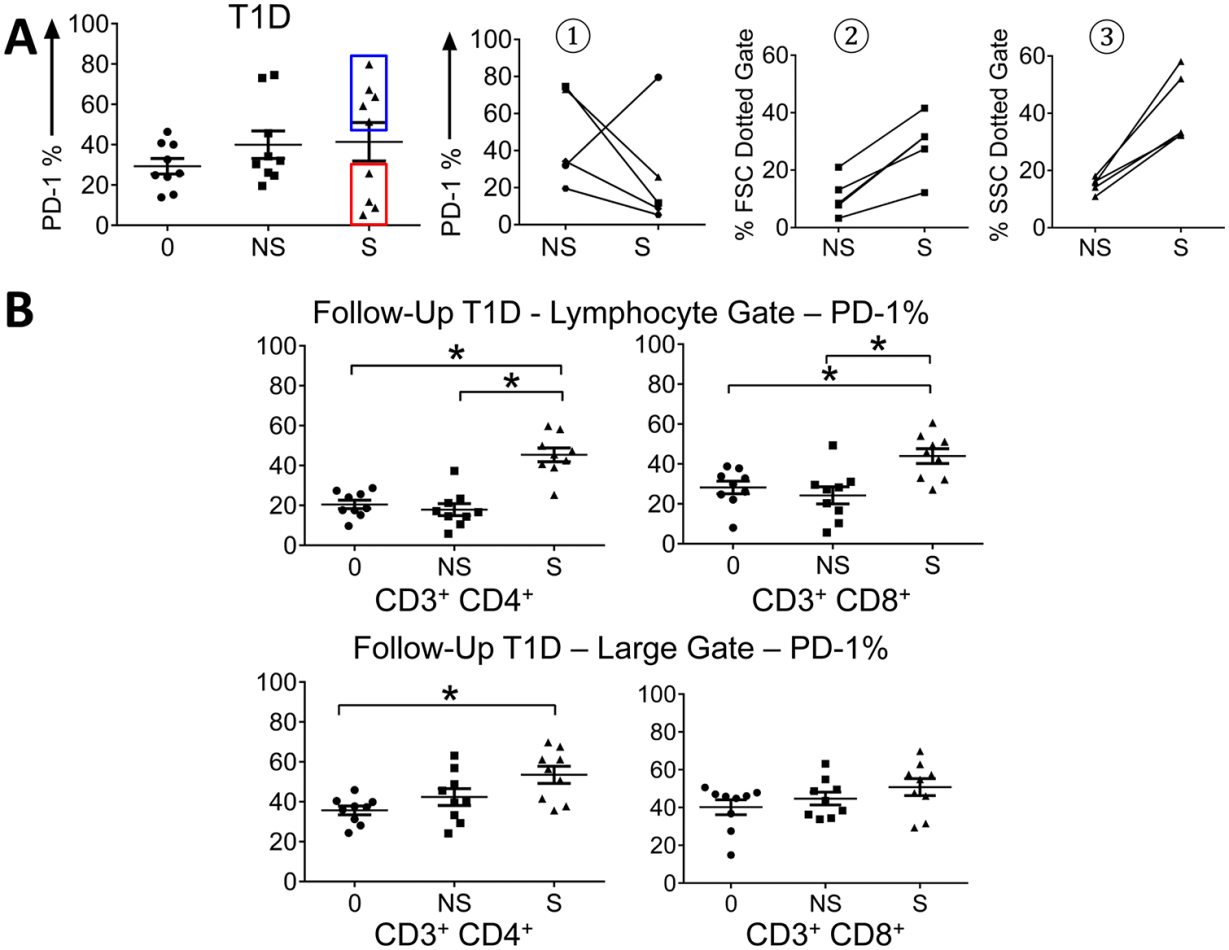
PD-1 expression recovers in T1D follow up. **A**. Subpopulations of T cells, one that down regulates PD-1 and the other that upregulates PD-1 **B**. Expression of PD-1 in T cells from T1D after 4–6 months post diagnosis (T1D Follow up) (* indicate p < 0.05). Top left CD3^+^ CD4^+^ lymphocyte gate, top right CD3^+^ CD8^+^ lymphocyte gate, bottom left CD3^+^ CD4^+^ large gate, and bottom right CD3^+^ CD8^+^ large gate.

Although on average the percentage of PD-1 expression in T1D is reduced upon stimulation compared to other study groups (Fig 1) there were 2 subpopulations of T cells (Fig 2 panel A), one that down regulates PD-1 and the other that upregulates PD-1. Interestingly, the expression of PD-1 in T cell subsets became normal in the 8 children with T1D Follow up (Fig 2 panel B). Thus, uncoupled PD-1 upregulation is not permanent but recoverable after insulin therapy.

### Cytokine analysis on supernatants obtained from PBMCs reveals no correlation with PD-1 expression

**Fig 3:**
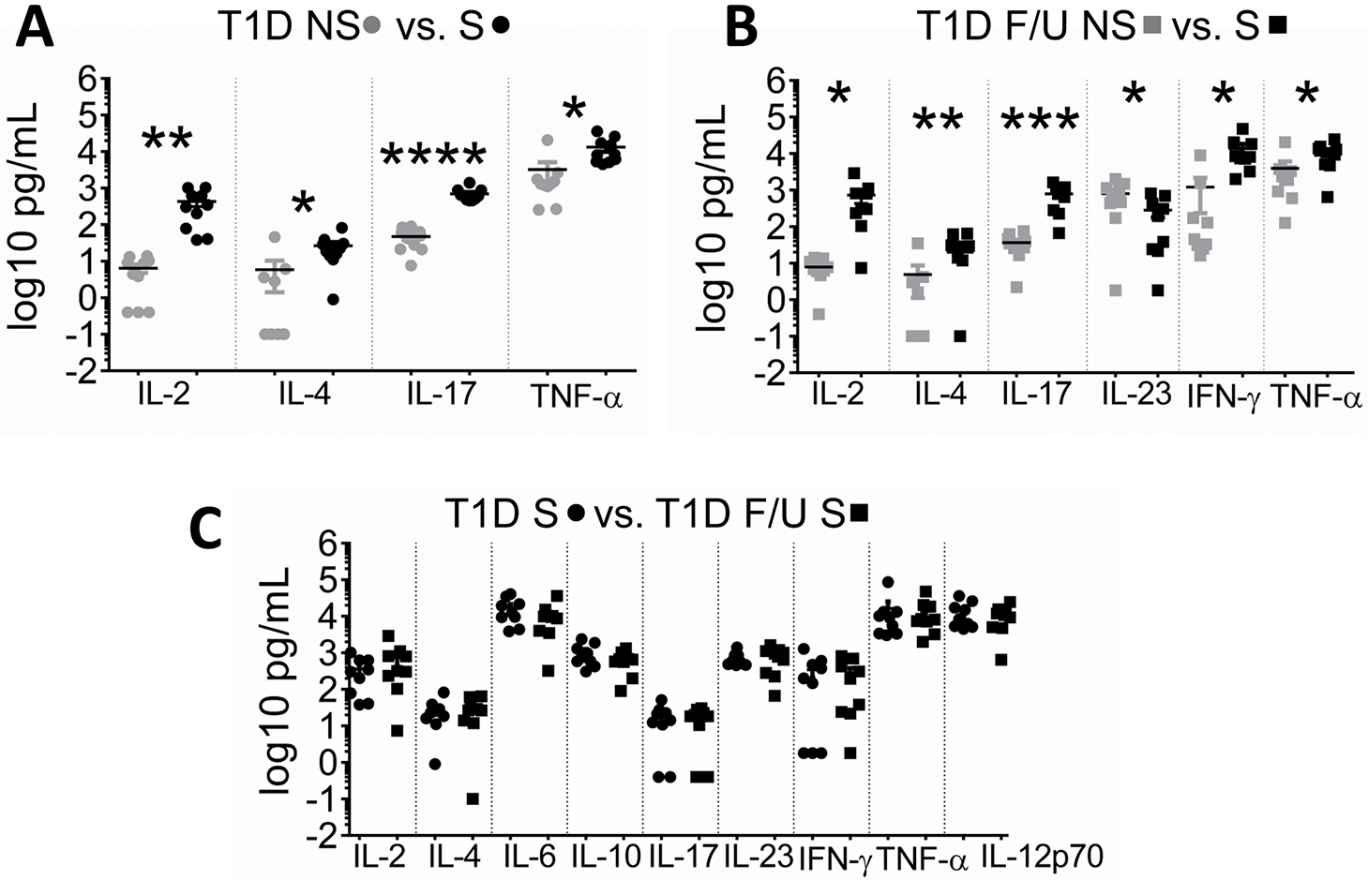
Cytokine production is not increased in T1D compared to T1D follow up. Cells were cultured for 24 h with and without stimulation with anti CD3/CD28, the supernatant collected and assayed by multiplex cytokine ELISA. Data were log-transformed to obtain normal distributions. **A. T1D NS (grey circles) vs S (black circles)** Cytokines that showed statistically significant different in T1D between unstimulated and stimulated PBMCs. **B. T1D F/U NS (grey circles) vs S (black circles)** Cytokine concentration in PBMCs from T1D Follow up. **C. T1D S (black circles) vs T1D F/U S (black squares)** Comparison of cytokine expression on stimulated PBMCs from subjects with T1D and T1D Follow up. (* indicate p < 0.05).

We performed subsequent experiments with PBMCs and not T cells due to the limited T cell number that could be obtained from the volume of whole blood drawn from our enrolled subjects. PBMCs from T1D responded to anti-CD3/-CD28 stimulation by potently increasing cytokine production measured in cell culture supernatants, both at diagnosis (T1D, Fig 3 panel A) as well as in follow-up (T1D Follow up, Fig 3 panel B). However, no differences in cytokine production were detected between stimulated T1D and T1D Follow up PBMCs (Fig 3 panel C). We also investigated if cytokine expression was different in the subjects that down regulated PD-1 at diagnosis onset compared against the subjects that upregulated PD-1 but found no differences in cytokines between these 2 subpopulations (not presented). This observation is evidence against our hypothesis that decreased PD-1 expression leads to over expression of pro-inflammatory cytokines by PBMCs, although unmeasured cytokines may have shown differences.

### PBMCs of T1D follow up have increased glycolysis

**Fig 4:**
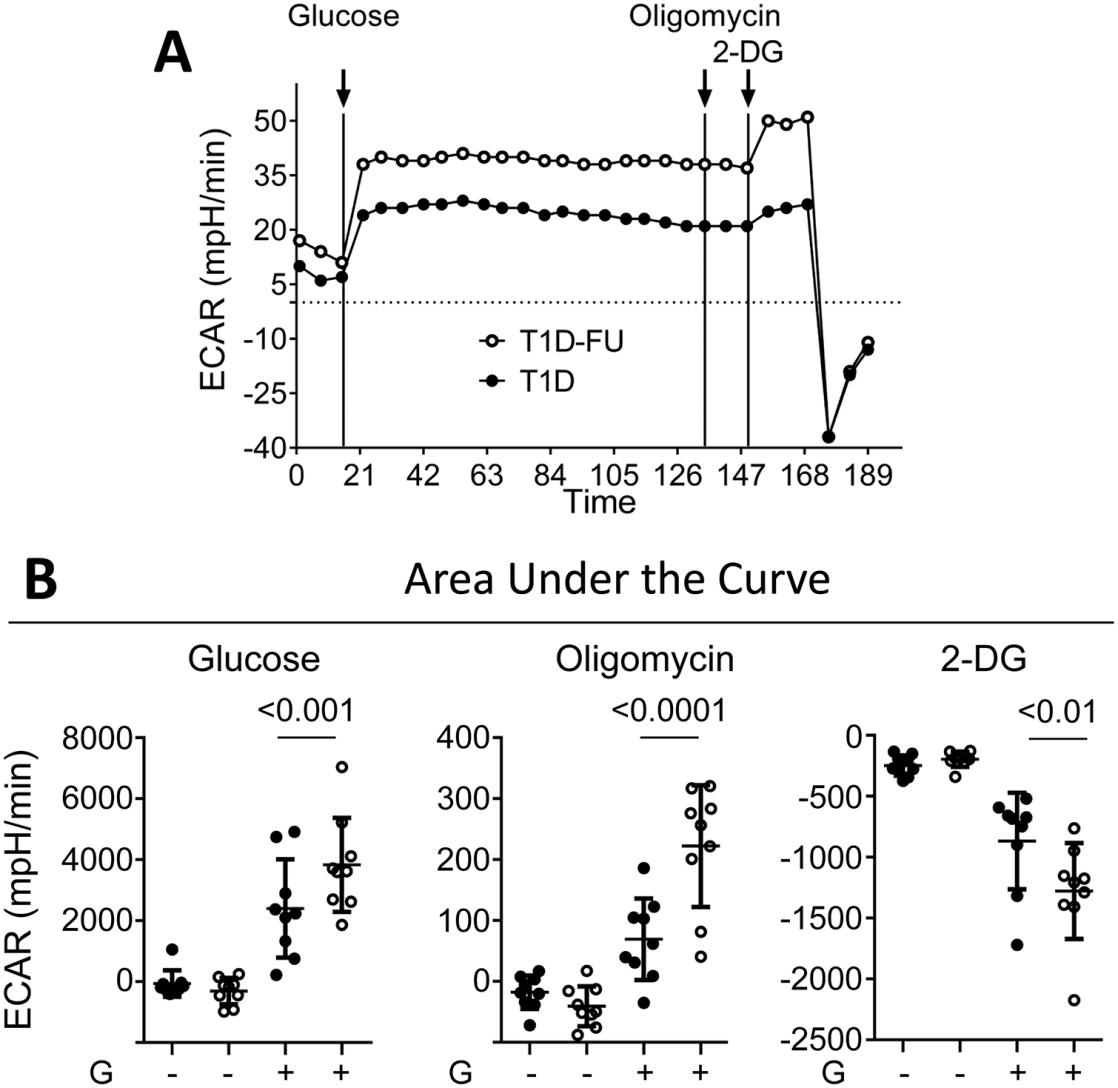
PBMCs from T1D follow up had enhanced glycolytic capacity compared to T1D. **A**. ECAR (mpH/min) in PBMCs from T1D (black circles) and T1D Follow up (open circles) after adding glucose, oligomycin and 2-DG. **B**. Integration of area under the curve showing comparisons in ECAR between T1D and T1D Follow up with additives, as shown.

To further explore the recently described relationship between PD-1 down regulation and glucose metabolism [10], we measured ECAR in PBMCs in response to glucose. The limitations in cell number did not allow us to sort and separate different cell subpopulations in order to assess the metabolic function of specific cells. Interestingly, however, we found that PBMCs from T1D follow up had enhanced glycolytic capacity compared to T1D (Fig 4 panel A) and CTRL (not presented). This pattern suggests that after 4–6 months of insulin therapy, PBMCs from T1D become hypermetabolic, at least as far as glucose consumption is concerned. We looked for differences in ECAR between PBMCs from T1D that down regulated and upregulated PD-1 upon stimulation. However, we found no significant differences between these groups (not shown).

## Discussion

Our study documents the dysregulation of membrane PD-1 in stimulated T cells in children with new onset T1D, it’s re-programming to normalcy after therapy with insulin and increased glycolytic capacity in PBMCs from T1D follow-up. Although based solely on our data we cannot conclude that a decreased expression of PD-1 in TCR-stimulated T cells participates in the pathogenesis of T1D, failure to upregulate PD-1 expression in T1D may contribute to the autoimmune state early in its clinical course [6, 8, 12, 13, 14, 15]. On the other hand, 4–6 months after diagnosis, we observed a recovery of expression of PD-1 in T1D follow up to levels seen in CTRL and CD. It is possible that by then pancreatic **β** cells have been completely obliterated, resulting in restoration of immune homeostatic mechanisms. Our findings therefore suggest that at the time of clinical diagnosis, T cells in T1D exhibit features of immune exhaustion.

Our observation that a subset of children with T1D down regulated PD-1 expression in T cells upon stimulation is intriguing. It is not yet known what factor(s) may contribute to the lack of upregulation of PD-1 in a subset of children with T1D. The alterations in PD-1 expression that we report could be a consequence of the metabolic state of T cells in T1D and to the lack of insulin at the time of diagnosis. While we observed no differences in cytokine output in stimulated PBMCs between children that up or down regulated PD-1, it is possible that “PD-1 downregulators” at diagnosis may be at risk for associated autoimmune complications later in the course of T1D, such as thyroid disease, celiac disease and Addison disease. This possibility should be investigated with a larger sample size and longer follow-up.

One unexpected finding was that upon follow up, PBMCs of T1D demonstrate an enhanced glycolytic capacity after institution of insulin therapy. This pattern suggests that PBMCs from T1D follow up are hypermetabolic post-diagnosis, which is a novel finding that is consistent with a greater capacity for cell division. Nevertheless this study does not directly assess T cells versus other immune cells due to limitations in collection of cell number, but at least this work sets the stage for future work to test metabolic “fitness” in different populations.

Although this was a pilot study with a limited sample, studying a pediatric population strengthens our work because in general, children lack comorbidities such as smoking, medications and alcohol use that may affect immune responses. Their caregivers supervise their medical treatment, which improves adherence to insulin therapy [16]. We aimed to match the age of children in all study groups to avoid possible confounding by the different stages of development of the immune system in different age brackets. Importantly, we had access to children with newly diagnosed T1D after metabolic stabilization (e.g., no ketoacidosis) and untreated children with new CD for comparison. Interestingly, T cells from children with CD significantly upregulated PD-1 in response to TCR engagement. While CD and T1D share some common immune mechanistic components [17]. the dysregulation in PD-1 expression on PBMC highlights important differences between the two diseases and underscore that CD is not a classic autoimmune disease [18].

Further work is needed to fully understand the PD-1 pathway and its relationship to metabolism at each phase of T1D. Using a diabetes-specific specific stimulus (e.g., islet peptide, insulin) to stimulate T cells to assess the expression of PD-1 is a next step. Studying a larger number of patients with T1D would give us more precise information about the contribution of this important regulatory pathway. We did not explore PD-L1 status due to constraints in cell numbers obtained from a limited volume of peripheral blood.

The PD-1 pathway may be targeted for novel therapies for prevention and immunomodulation of autoimmunity. For example, stem cells that act via the PD-L1 pathway suppress diabetogenic T-cell proliferation, reversing experimental type 1 diabetes. This work also provides insight into potential mechanisms by which the PD1—PD-L1 pathway supresses autoreactive T cells. [19, 20]. Fiorina et all have postulated that PD-1-PD-L1 play a principle role in the tolerogenic effect of hematopoietic stem cells and prolongs islet allograft survival via PD-L1 [21]. Children with T1D and dysregulated PD1 expression may be more susceptible to autoimmune complications of T1D, such as celiac disease and thyroiditis. Also, understanding how or why PD1 was not upregulated in T cells from a subpopulation of children with T1D may help develop new approaches to suppressing PD1 in specific T cells in the context of treatment for cancer [11].

Overall our data show a stronger link between metabolic dysregulation and PD1 expression in T cells in T1D, compared to cytokine production. This further supports the notion that PD1 expression may be a key pivot point for the understanding of the interplay between glucose metabolism and the immune response of T1D.
